# Effect of Dietary Sugar Intake on Biomarkers of Subclinical Inflammation: A Systematic Review and Meta-Analysis of Intervention Studies

**DOI:** 10.3390/nu10050606

**Published:** 2018-05-12

**Authors:** Karen W. Della Corte, Ines Perrar, Katharina J. Penczynski, Lukas Schwingshackl, Christian Herder, Anette E. Buyken

**Affiliations:** 1 Public Health Nutrition, University of Paderborn, 33098 Paderborn, Germany; s7kaward@uni-bonn.de (K.W.D.C.); katharina.penczynski@uni-paderborn.de (K.J.P.); 2 Nutritional Epidemiology, University of Bonn, DONALD Study, 44225 Dortmund, Germany; iperrar@uni-bonn.de; 3 Department of Epidemiology, German Institute of Human Nutrition, 14558 Nuthetal, Germany; Lukas.Schwingshackl@dife.de; 4 Institute for Clinical Diabetology, German Diabetes Center, Leibniz Center for Diabetes Research at Heinrich Heine University Düsseldorf, 40225 Düsseldorf, Germany; Christian.Herder@ddz.uni-duesseldorf.de; 5 German Center for Diabetes Research (DZD), 85764 München-Neuherberg, Germany

**Keywords:** dietary fructose, high fructose corn syrup, dietary sucrose, dietary glucose, inflammatory markers

## Abstract

It has been postulated that dietary sugar consumption contributes to increased inflammatory processes in humans, and that this may be specific to fructose (alone, in sucrose or in high-fructose corn syrup (HFCS)). Therefore, we conducted a meta-analysis and systematic literature review to evaluate the relevance of fructose, sucrose, HFCS, and glucose consumption for systemic levels of biomarkers of subclinical inflammation. MEDLINE, EMBASE, and Cochrane libraries were searched for controlled intervention studies that report the effects of dietary sugar intake on (hs)CRP, IL-6, IL-18, IL-1RA, TNF-α, MCP-1, sICAM-1, sE-selectin, or adiponectin. Included studies were conducted on adults or adolescents with ≥20 participants and ≥2 weeks duration. Thirteen studies investigating 1141 participants were included in the meta-analysis. Sufficient studies (≥3) to pool were only available for (hs)CRP. Using a random effects model, pooled effects of the interventions (investigated as mean difference (MD)) revealed no differences in (hs)CRP between fructose intervention and glucose control groups (MD: −0.03 mg/L (95% CI: −0.52, 0.46), *I*^2^ = 44%). Similarly, no differences were observed between HFCS and sucrose interventions (MD: 0.21 mg/L (−0.11, 0.53), *I*^2^ = 0%). The quality of evidence was evaluated using Nutrigrade, and was rated low for these two comparisons. The limited evidence available to date does not support the hypothesis that dietary fructose, as found alone or in HFCS, contributes more to subclinical inflammation than other dietary sugars.

## 1. Introduction

Chronic, low-grade inflammation is a key factor in the pathogenesis of cardiovascular disease [1], and is associated with the risk of developing diabetes [2,3], dementia [4], and depression [5]. Also, low-grade inflammation is related to a higher risk of all-cause mortality in old age [6]. Therefore, identifying modifiable risk factors that could effectively lower chronic inflammation would contribute to the prevention of chronic disease.

According to observational data reports, it has been consistently reported that dietary sugar intake (more specifically, sugar-sweetened beverages (SSB)) may be one stimulus of subclinical inflammation, as measured by the inflammatory marker C-reactive protein (CRP) [7,8,9,10]. Dietary sugar is consumed in significant amounts in Western diets. In a review of the sugar consumption of 18 developed countries, it was found that total sugar intake as a percentage of energy ranged between 13.5–24.6% in adults [11]. In the United States, Nationwide Food Consumption Surveys (NHANES) have suggested that the percentage of sweeteners from high-fructose corn syrup (HFCS) increased from 16% in 1978 to 42% in 1998, and then stabilized [12]. A similar trend pattern was also observed for total fructose intake as a percentage of carbohydrates [13]. The most recent data has shown that with increased public awareness, the consumption of added sugar in the United States has actually decreased between 1999 and 2008, from a mean of 18.1% of total energy to 14.6% [14]. Overall sugar energy intakes are, however, still much higher than the United Kingdom’s Scientific Advisory Committee on Nutrition (SACN) guidelines, which recommend a maximum free sugars intake of 5% daily energy intake [15], and the World Health Organization (WHO), which recommends a maximum of 10% (5% for further health benefits) [16].

It has been postulated that dietary sugar consumption contributes to increased inflammatory processes in humans. Central to the potentially relevant mechanisms is the fact that dietary sugar promotes de novo synthesis of free fatty acids (FFA) in the liver [17,18,19], which according to the lipotoxicity theory, would produce FFA metabolites that may trigger inflammatory processes and reactive oxygen species (ROS) formation [20,21].

The differences in the metabolism of fructose (alone or found in sucrose) versus that of glucose should be considered, in order to distinguish what potential role these monosaccharides may play in increasing inflammatory processes. In contrast to glucose, which can be metabolized by any cell in the body, fructose must be metabolized in the liver. Since there are no negative feedback mechanisms that control for and prevent excess substrate supply of fructose to liver mitochondria, fructose is independently partly converted to acetyl-CoA, which is a building block for fatty acid synthesis [19]. This metabolic pathway of fructose supports the lipotoxicity theory, however, it remains to be established whether dietary fructose/sucrose is more important than dietary glucose for promoting inflammation in human studies.

Therefore, the aim of the current meta-analysis and systematic review was to evaluate the evidence from published human interventional studies regarding the relevance of dietary fructose (alone, or found in sucrose or HFCS) and dietary glucose as a comparator for biomarkers of subclinical inflammation. This evaluation was done quantitatively, through a meta-analysis, and qualitatively, through a brief narrative review. Quality of meta-evidence was also assessed [22]. We selected the acute-phase protein high-sensitivity C-reactive protein (hsCRP), proinflammatory cytokines (interleukin-6 (IL-6), interleukin-18 (IL-18), interleukin-1 receptor antagonist (IL-1RA), tumor necrosis factor-α (TNF-α)), the chemokine monocyte chemoattractant protein 1 (MCP-1), soluble adhesion molecules (soluble E-selection (sE-selectin), soluble intercellular adhesion molecule-1 (sICAM-1)), and the anti-inflammatory adipokine adiponectin as biomarkers of subclinical and vascular inflammation, because they are the most commonly measured inflammation-related biomarkers in clinical and epidemiologic studies, with established associations with cardiometabolic diseases [23,24,25,26,27,28].

## 2. Materials and Methods

### 2.1. Study Selection

**Figure A1:**
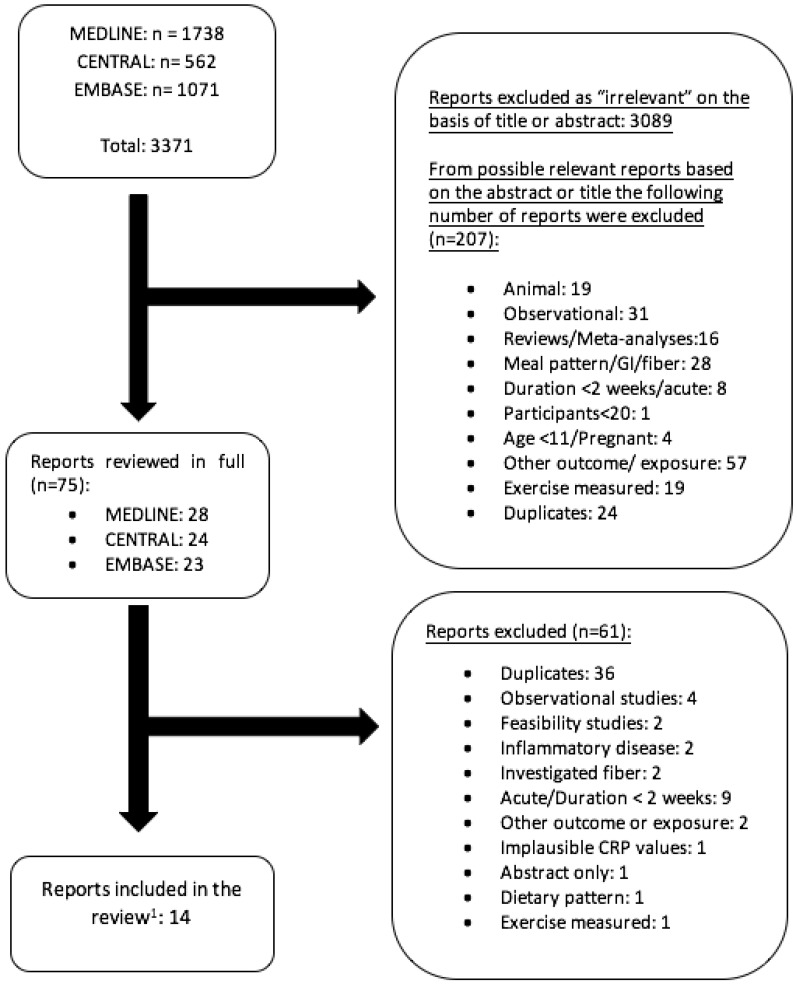
Study selection process. ^1^ Of the 14 studies, two reports from one original study by Stanhope et al. [32] and each study (Cox et al. [33] Rezvani et al. [34]) report on different inflammatory markers measured in the original study. Therefore, there are technically 13 studies included in the review.

We conducted a systematic literature search of the MEDLINE (http://www.ncbi.nlm.nih.gov/pubmed/), EMBASE (https://www.elsevier.com/solutions/embase-biomedical-research), and Cochrane Library (Cochrane Central Register of Controlled Trials (CENTRAL)) (http://onlinelibrary.wiley.com/cochranelibrary/search/) databases from January 1990 through 18 April 2018. The search was limited to this time frame because hsCRP assays and sensitive assays for low-abundance cytokines, such as IL-6, were not available before this time period. This review was registered with PROSPERO (registration number: CRD42017081171). The guidelines found in the Cochrane handbook for systematic reviews of interventions were used in writing this review [29]. The following terms were used to identify all potentially relevant publications published in the English language: (dietary) sucrose, glucose, and fructose/HFCS together with (high sensitivity) C-reactive protein, ((hs-)CRP), interleukin 6 (IL-6), interleukin 18 (IL-18), interleukin-1 receptor antagonist (IL-1RA), tumor necrosis factor-α (TNF-α) monocyte chemoattractant protein 1 (MCP-1)/CCL2, E-selectin, intercellular adhesion molecule 1 (ICAM-1) or adiponectin (for details on the search terms used see “Supplemental Data”). The search was restricted to human intervention studies (controlled, parallel, or crossover design). Inclusion criteria limited search to either (1) healthy, overweight, or obese adults or adolescents (age 11 and up), (2) with or without diseases for which inflammation is not a major symptomatic factor. Inclusion criteria further limited search to studies in which (3) dietary fructose, glucose, or sucrose was administered as predictors (including information on intake amounts of respective sugars), and with (4) C-reactive protein (CRP); the proinflammatory cytokines IL-6, IL-18, and TNF-α; the anti-inflammatory IL-1RA; the chemokine MCP-1/CCL2, the soluble adhesion molecules ICAM-1 and E-selectin; and adiponectin as outcome measures. A detailed listing of all inclusion and exclusion criteria was included in our PROSPERO registration. Because we were interested in the specific effects of dietary fructose, sucrose, and glucose on low-grade inflammation, we excluded studies that analyzed dietary patterns, effects of glycemic index (GI), treatment studies, or studies on pregnant women (for n-numbers, see Appendix A Figure A1). Studies that assessed the effects of fiber intake simultaneously with sugar intake were also excluded. We additionally excluded intervention studies on participants with major inflammatory diseases, such as arthritis, hepatitis, or irritable bowel syndrome, i.e., diseases for which inflammation or oxidative stress with clinical symptoms are major symptomatic factors in their progression or development.

Furthermore, because we were interested in investigating the potential impact of dietary sugars on chronic inflammation, rather than short-term responses to diet, we chose to include only studies with a duration of at least 2 weeks. The literature search was conducted independently by two investigators (K.W.D.C. and I.P.). The study selection process is illustrated in Appendix A Figure A1.

### 2.2. Data Extraction

**Figure A2:**
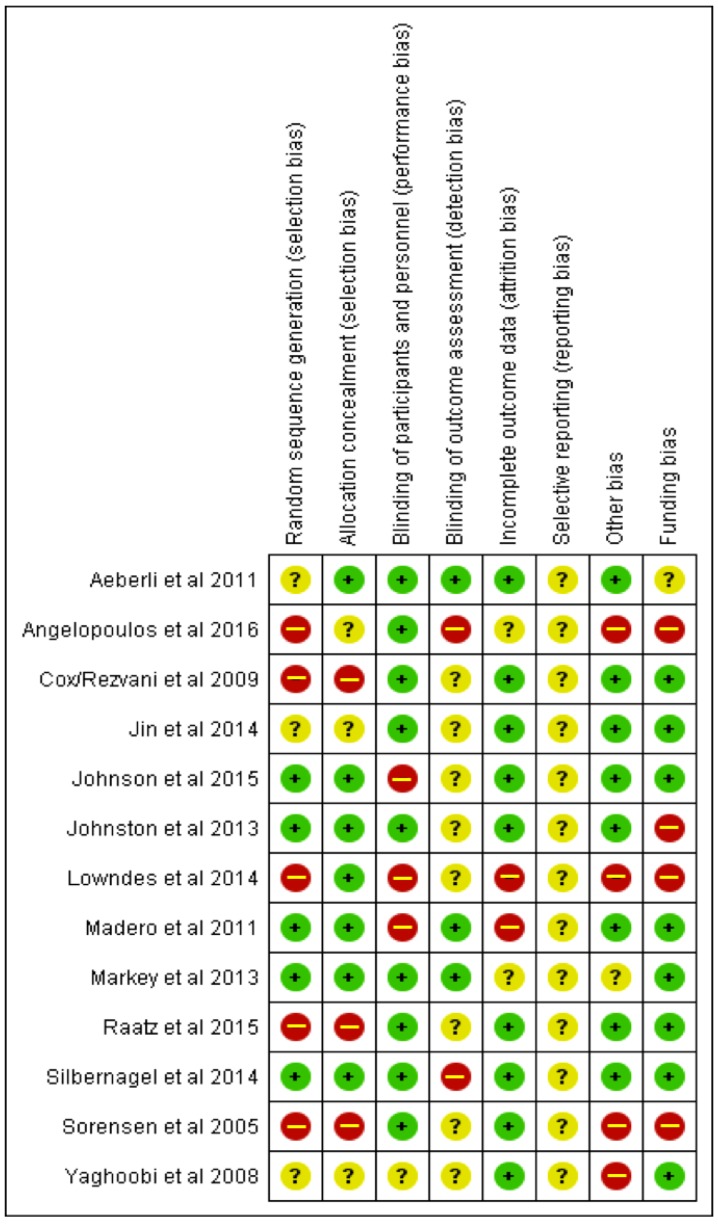
Risk of bias table.

Two investigators (K.W.D.C. and I.P.) independently reviewed and extracted relevant data from each report. Extracted data included information on study design, duration, location, sample size, participant characteristics (sex, age, BMI, health status), type of intervention, and dietary sucrose, fructose, HFCS, or glucose intake amounts (see Table 1 and Table 2. If available, data on CRP, IL-6, IL-18, TNF-α, IL-1RA, MCP-1, sICAM-1, sE-selectin, and adiponectin were extracted as the biomarkers of subclinical inflammation of interest. We extracted data from baseline and change and/or endpoint of these outcome measurements, in order to calculate percent changes to include into the tables describing the studies. Post-intervention means, standard deviations, and number of participants were collected, and the statistical software tool Review Manager 5.3 (Nordic Cochrane Center, Copenhagen, Denmark) was used for the statistical analyses. Where post-intervention means were not available, change from baseline data was extracted. A comparator subgroup (e.g., free fructose vs free glucose) was only included in the meta-analysis if there were at least three studies available to report on. Information on whether diets were hypercaloric, eucaloric, hypocaloric, or isocaloric was also extracted. Hypercaloric diets were determined to be those in which energy intakes were ad libitum and unregulated, in addition to offering sugar-sweetened liquids or foods that caused an increase in energy intake compared to baseline. Implausible data were corrected and confirmed by contacting original authors in one case. Data were also extracted for the purposes of conducting a bias assessment using the Cochrane Collaboration’s assessment tool to elucidate the risk of bias and attached either a low, unclear, or high risk of bias in eight areas to each study (see Appendix A Figure A2). 

### 2.3. Statistical Analysis

Investigation of the effects of dietary sucrose, fructose, glucose, or HFCS on biomarkers of subclinical inflammation was done using a random effects model. In this model, post-intervention mean values and corresponding standard deviations (if not available, standard errors or 95% confidence intervals were used to calculate the standard deviation) for intervention and control groups were pooled. As recommended by the Cochrane Handbook, we extracted post-intervention means where possible. If they were not available, change from baseline values were extracted [29]. As the main outcome to be analyzed in the meta-analysis, pooled effects of the different interventions were investigated as mean difference (MD) by subtracting control group mean values from intervention group mean values. A standard χ^2^ test was used to test the heterogeneity between trial results. To measure inconsistency between study results, the *I*^2^ parameter was used: *I*^2^ = 100% × (*Q* − df)/*Q*, where *Q* is the χ^2^ statistic and df is its degrees of freedom [30]. The observed numerical value for *I*^2^ depends on the direction and magnitude of the effect, and the strength of evidence for heterogeneity (e.g., confidence interval for *I*^2^ or *p*-value from the chi-squared test) [29]. An *I*^2^-value of greater than 50% was considered to indicate substantial heterogeneity [31].

### 2.4. Assessment of Quality of Meta-Evidence

To evaluate the quality of meta-evidence for the association between dietary sucrose, fructose, glucose, and HFCS on subclinical inflammation we applied the NutriGrade scoring system [22]. NutriGrade comprises the following items for meta-analysis of randomized controlled trials: (0–10 points): (1) risk of bias (2) precision, (3) heterogeneity, (4) directness, (5) publication bias, (6) funding bias, (7) and study design. Based on this scoring system, four categories were recommended to judge the meta-evidence: high (≥8 points), moderate (6 to 7.99 points), low (4 to 5.99), and very low (0 to 3.99) [22].

## 3. Results

### 3.1. Description of Studies

The literature search was conducted on 18 April 2018. The detailed steps of the systematic search and selection process are given as a flow diagram (see Appendix A Figure A1). Taken together, 13 studies were identified by the search as matches, and were included in the review (one study [32] resulted in two publications: Cox et al. [33] and Rezvani et al. [34], each covering different outcomes). Of these 13 studies, 8 addressed fructose, 3 HFCS, 7 sucrose, and 6 glucose (see Table 1 and Table 2).

The 13 intervention trials that addressed dietary fructose, sucrose, glucose, or HFCS intake as a nutritional exposure variable [32,33,34,35,36,37,38,39,40,41,42,43,44,45,46] lasted between 2 weeks and 3 months, and included a total of 1141 participants (range: 20–355), aged 11–72 years, with a BMI ranging from 19 to 40 kg/m². Two studies were performed on men only [37,39], and one study included women only [40]. The dietary fructose intake ranged from 17 grams daily to 217 grams daily in the eight fructose intervention studies. Six of the 8 studies on fructose compared free fructose to free glucose isocalorically. The other two fructose studies investigated the effects of low-fructose, hypocaloric diets. Dietary sucrose intake for the intervention groups in the seven sucrose studies ranged from 50 to 203 g of daily intake. These sucrose studies had diverse exposures in the control groups, which they compared to dietary sucrose intake. Three of them compared sucrose-sweetened beverages to glucose-, fructose-, or HFCS-sweetened beverages in isocalorically matched or differing intake amounts [38,39,43]. Two of these three administered sugars in a milk medium: sucrose-sweetened low-fat milk was compared to HFCS-, fructose-, and glucose-sweetened low-fat milks in Angelopoulos et al. [38] and sucrose-sweetened milk was compared to HFCS-sweetened milk in Lowndes et al. [43].

Two studies compared sucrose to either an artificial sweetener control group [46] or to sugar-reformulated products [45], two additional sucrose intervention studies had either a honey-intake control group [44], or both fructose and honey control groups [42].

### 3.2. Meta-Analysis Results

Owing to the different designs of the intervention trials, they were classified in subgroups for the meta-analysis according to the types of dietary sugar interventions and controls as follows (Table 3, *n* = number of studies in each subgroup):

- (a) Fructose vs glucose (*n* = 6 studies; *n* = 8 study arms);
- (b) HFCS vs sucrose (*n* = 3 studies; *n* = 6 study arms).

The analyses of the comparators high-fructose vs low-fructose, fructose vs sucrose, and glucose vs sucrose could not be performed because these subgroups had two or less comparisons to report on. For the same reason, the outcomes of IL-6, MCP-1, sICAM1, sE-selectin, adiponectin, and TNFα were not included in the meta-analysis. No studies were found that reported effects of dietary sugar on IL-18 or IL-1RA.

(a) Fructose vs glucose

**Figure A3:**
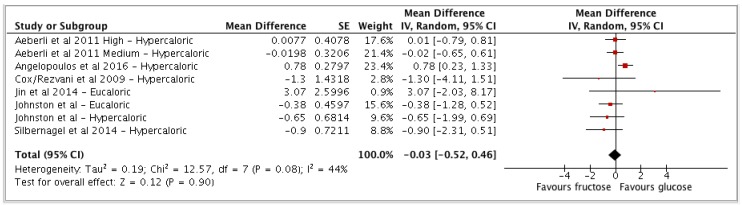
Forest plot for fructose vs glucose and (hs)CRP as an outcome. Pooled estimates of effect sizes (95% confidence intervals) for the comparison of fructose and glucose expressed as mean differences (MD). Effect size units expressed in mg/L. MD = mean of intervention group – mean of control group. Fructose = intervention group and glucose = control group. No difference in effects on (hs)CRP between fructose and glucose groups. Tau^2^ value represents degree of heterogeneity within comparison groups. *I*^2^ represents the extent to which studies are statistically inconsistent. “Hypercaloric” refers to those trials that administered extra-physiological doses of sugars and “eucaloric” to trials in which daily energy requirements were not exceeded or restricted by administered doses of sugars.

The comparator of fructose interventions vs glucose control groups showed no differences for CRP (MD: −0.03 mg/L 95% CI −0.52 to 0.46, *I*^2^ = 44%) (see Appendix A Figure A3).

(b) HFCS vs sucrose

**Figure A4:**
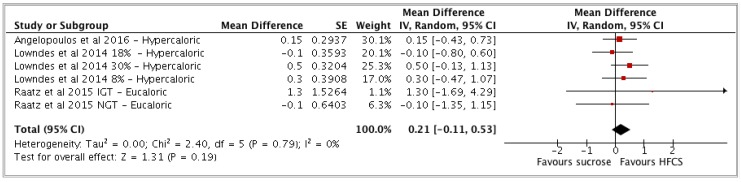
Forest plot for HFCS vs sucrose and (hs)CRP as an outcome. Pooled estimates of effect sizes (95% confidence intervals) for the comparison of HFCS and sucrose expressed as mean differences (MD). Effect size units expressed in mg/L. MD = mean of intervention group − mean of control group. HFCS = intervention group and sucrose=control group. The increase in (hs)CRP was greater in HFCS groups vs sucrose groups but not significant. Tau^2^ value represents degree of heterogeneity within comparison groups. *I*^2^ represents the extent to which studies are statistically inconsistent. “Hypercaloric” refers to those trials that administered extra-physiological doses of sugars and “eucaloric” to trials in which daily energy requirements were not exceeded or restricted by administered doses of sugars.

In the comparator of HFCS interventions vs sucrose control groups, changes in CRP were non-significantly higher in HFCS compared to sucrose groups (MD: 0.21 mg/L 95% CI -0.11 to 0.53, I^2^= 0%) (see Appendix A Figure A4, Table 3).

### 3.3. NutriGrade

Overall, the quality of meta-evidence for the association between dietary fructose vs glucose, and comparing HFCS vs sucrose on CRP was rated as “low”, (i.e., confidence in the effect estimate is low). Further research will provide additional evidence, and will likely change the effect estimate. This judgement was mainly based on the low number of identified studies and study participants, the study limitations, and the imprecise effect estimates.

### 3.4. Comprehensive Narrative Overview

Due to the fact that the quality of meta-evidence as assessed by NutriGrade was low, the studies were heterogeneous in nature, and only a portion of the studies were able to be quantitatively assessed by the meta-analysis, an additional narrative review may provide further insights not captured by the meta-analysis.

The six studies included in the meta-analysis compared free fructose intake intervention groups with free glucose intake control groups isocalorically [32,33,34,35,36,37,38,39], and therefore, warrant further scrutiny. Two of these studies reported significant effects on biomarkers of subclinical inflammation: Jin et al. [35] found a difference between the fructose and glucose group in hsCRP (increase by 4% vs decrease by 23%, respectively). Cox/Rezvani et al. (both reporting from the original study by Stanhope et al. [32]) reported a treatment effect confined to MCP-1, where values in the fructose group increased by 38%, while decreasing in the glucose group by 9%. No treatment effects were seen for hsCRP, IL-6, sE-selection, or adiponectin in this study. While Aeberli et al. [39] did not observe a significant overall effect, hsCRP increased in all interventions (by 82–109%) comparing varying amounts of fructose, glucose, or sucrose with the greatest increase in the high-fructose and the high-sucrose group (109% and 105%, respectively). In the study by Angelopoulous et al. [38], increases in CRP were confined to the fructose and the glucose interventions, both amounting to approximately 24%, yet overall effects did not differ between the four intervention groups. The study by Johnston et al. [37] did not show any significant differences between the high-fructose and high-glucose groups for CRP and IL-6 outcomes. Silbernagel et al. [36] did not report significant differences between the glucose or fructose intervention groups, yet a substantial increase of 57% was only found for CRP in the glucose group.

The additional two studies on fructose that were not included in the meta-analysis used a low-fructose, [40,41] low-calorie diet, and measured the effects on CRP and sICAM values, which decreased in both intervention and control groups. Sorensen et al. [46] conducted a 10-week long study and reported an insignificant difference in CRP concentrations in the sucrose-added group (increase of 6%) and a decrease in the artificial sweetener group (decrease of 26%). None of the other intervention studies reported an effect of sucrose on the investigated biomarkers of subclinical inflammation. Of note, in the study by Angelopoulos et al., the fructose- and glucose-intake groups reported a 24% increase in CRP concentrations, while the sucrose-intake group experienced no increase [38].

## 4. Discussion

The current systematic review and meta-analysis identified 13 intervention studies that addressed the relevance of dietary fructose, sucrose, and glucose for biomarkers of subclinical inflammation, as assessed by (hs)CRP, IL-6, TNF-α, MCP-1, sE-selectin, sICAM-1, and adiponectin. Pooled effects of the different interventions (investigated as mean differences) revealed that fructose intervention groups showed no significant differences in (hs)CRP when compared to glucose control groups. Similarly, no differences were observed between HFCS vs sucrose interventions. As summarized narratively, effects were observed in a small number of studies, but the overall picture is inconsistent, the effect sizes are variable, and the overall quality of meta-evidence is low. Additional evidence from future intervention studies on this topic are needed in order to draw confident conclusions as to the effects of dietary sugar on subclinical inflammation.

### 4.1. Implications Arising from the Study Design

Evaluation of the impact of dietary sugars is often hampered by the lack of an isocaloric comparator, which makes it therefore unclear whether the effects result from fructose or sucrose itself, or simply excessive energy consumption [47]. A strength of most of the fructose intervention studies included in this review is that they do administer fructose with an isocaloric control group of dietary glucose, although most of these studies were hypercaloric trials. The fact that there was often no difference observed between intervention and control groups in studies that administered supra-physiological doses [32,36,37,38,43] may be due to post-prandial stress, that activates low-grade inflammation due to extremely high energy intake. If this is the case, then no conclusions can be drawn about the unique effects of fructose versus glucose metabolism from studies that administer supra-physiological doses across comparative groups. In order to be able to draw firm conclusions for health care policy decisions, more large-scale, effective, longitudinal intervention studies that investigate sucrose or HFCS ingested at average intake levels (75–100 grams/day) compared to control groups, as well as studies which ingest sucrose or HFCS at the lower levels recommended by WHO or the UK’s SACN guidelines (5% of energy intake or about 25 grams/day), need to be conducted with low-grade inflammation as an outcome measure. Since sugar is typically not consumed in an isolated monosaccharide form, studies which compare glucose and fructose isocalorically at average consumption levels would serve to further understand the unique metabolic differences between fructose and glucose, and how they affect inflammation.

The methodological limitations of the two largest studies both lasting 10 weeks are worth mentioning. These two studies used HFCS- [38] or sucrose-sweetened milk [43] as intervention diets, and addressed the effect on CRP only. Both studies did not specify how randomization was done (risk of selection bias) and had relatively high drop-out rates of 27% and 26%, respectively. In view of the risk of bias, the absence of effects reported from these studies should be interpreted with caution, also because these studies were either funded by the sugar industry or the authors received consulting fees from organizations that market or utilize fructose, HFCS, or sucrose. Results from industry- and non-industry-funded research often differ, and a recent analysis suggests that industry-funded research tends to underestimate the adverse effects of dietary sugar [48]. On the other hand, personal views from non-industry researchers could present another source of bias. Such bias, however, is difficult to address, because there is no marker for this potential problem.

Finally, some methodological aspects in the measurements of inflammatory markers can be considered. Ideally, one would like to have a clear picture of which biomarkers are responsive to fructose and sucrose across all studies, which would, in turn, allow conclusions on the pathways relevant for disease prevention. This is, however, hampered by methodological problems, ranging from the use of very different/non-standardized assays to the large inter-individual variation in inflammatory biomarkers. Such variation is affected by the presence or absence of accompanying metabolic conditions. Repeated measures both pre- and post-intervention would be required in order to ascertain the size of such variations in a study population.

### 4.2. Potentially Relevant Pathophysiological Mechanisms

Evidence from human intervention studies suggests that doses of fructose providing excess energy (+21–35% energy) raise liver fat [17,18]. This effect, however, appears to be confounded by excessive energy intake [17,18]. As previously discussed, the metabolism of dietary fructose (alone or found in sucrose) has been reported to promote de novo synthesis of FFA in the liver when consumed in high amounts [17,19]. While hepatic triglyceride storage and accumulation seem to be a benign symptom of steatosis, there is preliminary evidence that FFA metabolites may contribute to the progression of non-alcoholic fatty liver disease (NAFLD) to non-alcoholic steatohepatitis (NASH) by triggering inflammatory processes, ROS formation, and apoptosis [20,21]. Studies involving tissue biopsies indeed showed a gradual increase in systemic levels of biomarkers of inflammation, such as CRP, IL-6, and IL-1RA, from healthy adipose tissue to adipose tissue heavily infiltrated by immune cells [49], and from healthy livers to NASH [50]. Therefore, it is plausible to hypothesize that specifically supplementary fructose exerts adverse effects. Hence, future studies should use both outcome measures of hepatic fat/de novo lipogenesis as markers for NAFLD, while simultaneously measuring more specific inflammatory markers ideally related to hepatic fat, e.g., fetuin A, etc. There is some evidence from human and animal studies that suggests a relationship between fructose consumption and increased visceral adipose tissue [51], which is metabolically active and produces numerous inflammatory cytokines, including TNF-α, and IL-6 [52], which may, in turn, induce CRP release in the liver. In addition, other mechanistic animal studies suggest that fructose leads to intestinal bacterial overgrowth and increased intestinal permeability, which causes endotoxin levels of lipopolysaccharides to translocate and activate Toll-like receptor 4 in liver Kupffer cells [53,54], whose activation leads to the release of several cytokines, including mainly TNF-α [55]. In another animal study, Gersch et al. reported that a high-fructose diet significantly increased the renal expression of MCP-1 in rats [56]. An in vitro study in human epithelial tubular cells suggested a response of MCP-1 production induced by fructose, but not glucose [57].

Looking at the effects of glucose, there is evidence for a specific role of glucose on oxidative markers, which can, in turn, lead to increases in biomarkers of chronic inflammation. High dietary glycemic index (GI) has been linked to increased inflammatory responses by means of recurrent hyperglycemic responses in the early postprandial phase, as well as elevated levels of free fatty acids in the late postprandial phase, both of which are considered to result in an overproduction of free radicals and releases of proinflammatory cytokines, which may, in turn, induce inflammation and vascular damage [58]. Fructose has a low glycemic index (GI), and there is evidence that suggests that consumption of foods with a lower dietary GL/GI is associated with anti-inflammatory effects. Accordingly, ingestion of fructose may contribute to a reduction of chronic inflammation, due to the avoidance of glycemic spikes [59]. This may be one potential explanation for the absence of consistent signals or harm in response to fructose intake.

Because obesity and sugar intake are closely linked (at least with respect to SSB) on the one hand, and obesity and inflammation are closely linked on the other, it is certainly possible that weight gain is a potential mediator in the association between sugar intake and inflammation. In this context, it is of interest to separately investigate the eucaloric and hypercaloric effects of dietary sugars on inflammatory markers, as the former can give relevant information on the metabolic pathways linking sugar and inflammation, whereas the latter informs on the public health relevance for inflammatory biomarkers in a hypercaloric setting, as is often the case in real-world intakes. The question of whether it is excess energy intake or excess sugar intake that leads to adverse health outcomes requires further research. Similar work was done in a review by Sievenpiper et al., who found that fructose intake only affected weight gain in hypercaloric versus isocaloric trials [60].

Considering the relationship between obesity and low-grade inflammation, a discussion of the change in body weight in the included trials would be relevant. Two studies included in this review observed that weight loss resulting from energy-restricted diets was associated with greater improvements in inflammatory markers (regardless of fructose-intake amounts) [40,41]. The remainder of the studies did not take changes in weight into account. Future studies interested in the association between dietary sugar intake and low-grade inflammation should bear in mind that weight loss may mediate the results [61].

### 4.3. Interpretation in the Context of Evidence from Observational Studies

One could argue that our data disagree with evidence from observational studies, since the relationship between SSB intake and the inflammatory biomarker CRP has been consistently reported in observational studies [8,9,10]. SSB intake was also associated with IL-6 and TNF-α [7], and recent studies link SSB intake to the inflammatory disease rheumatoid arthritis [62,63]. Due to concerns that SSB intake could be a marker for an undesirable diet [64,65], there is dispute as to whether results from observational data linking SSB intake to adverse health outcomes are impacted by residual confounding, i.e., that failure to adjust for various (non-measured) lifestyle factors could lead to an overestimation of the strength of positive associations. Since, in practice, fructose and glucose are consumed together (HFCS, sucrose, sugar from fruits), even separate appraisals of fructose and glucose intake on inflammatory outcomes and/or liver outcomes in observational studies may provide only limited information on how these sugars contribute to health outcomes. Hence, it is not possible to state whether our results from intervention studies agree with observational data. 

### 4.4. Strengths and Limitations

The strengths of this meta-analysis and systematic review include its standard to only analyze controlled human intervention trials, and its inclusion of six interventions that had similar isocaloric control groups. The inclusion of a broad range of biomarkers of subclinical inflammation is also a strength, and allows for a more thorough evaluation of the effects of dietary sugar on low-grade inflammation. In analyzing the main findings, quantification of the observed associations was possible, and is a strength of this review. The objective quantification of the effects in this meta-analysis was reinforced by additionally carrying out a risk of bias assessment and performing an analysis on the quality of meta-evidence. The limitations of this review lie in the fact that the data have substantial heterogeneity, and some of the comparisons had only a small number of studies. The studies range in duration from two weeks to three months. Most of the fructose studies compared glucose to fructose isocalorically (*n* = 6), but comparators in the rest (*n* = 7) vary. Intake amounts of the investigated sugars varied widely as well. Most of the studies are generally similar in design, exposures, interventions, and outcome measurements, but there are several that do not compare easily. Two of the studies measured the effects of artificial sugars, which may have unique metabolic effects. The study populations differed from each other in age, body weight and health status. The included interventions had generally shorter durations, and therefore, do not reflect the overconsumption of fructose over years in the general population, as is better depicted in observation studies.

## 5. Conclusions

In conclusion, the overall findings, as collectively analyzed by a meta-analysis, do not support the hypothesis that dietary fructose (alone or in HFCS) is more detrimental with respect to subclinical inflammation than dietary glucose or sucrose. However, the studies included in this review were heterogeneous, and several of the comparisons had only small numbers of studies, providing limited evidence. Consequently, the grading of meta-evidence was low for all comparisons. In order to draw more confident conclusions about the proinflammatory effects of free fructose or fructose found in sucrose versus glucose, or other non-fructose containing carbohydrates (fructose vs glucose, sucrose, vs maltose, sucrose vs refined carbohydrates), further human intervention studies with larger sample sizes, longer follow-up periods, better controlled designs, and with subclinical inflammation as a priori planned outcome, are required.

## Figures and Tables

**Table 1:** Dietary intervention studies investigating the effect of fructose/HFCS, sucrose, or glucose on biomarkers of subclinical inflammation. Extracted data on participants’ characteristics, study designs, dietary interventions, form of sugar, and feeding control.

First Author, Year, Country	Study Design	Participants’ Characteristics	Duration (weeks)	Intervention	Energy Intake	Sugar form §	Feeding Control ^‡^
Aeberli et al. (2011) [39] Switzerland	Crossover Double-blind	29 healthy males Age 20–50 years BMI 19–25 kg/m^2^	3	Intervention: high-fructose (80 g/day) medium-fructose (40 g/day) high-sucrose (80 g/day) high glucose (80 g/day) medium glucose (40 g/day) Control: low-fructose diet (33 g/day)	Hypercaloric *	Liquid	Supp/DA
Angelopoulos et al. (2016) [38] USA	Parallel Double-blind Randomized	267 healthy participants (96 m/171 w) Age 37.7 ± 12.1 years BMI 26.3 ± 3.3 kg/m^2^	10	Intervention: sucrose-sweetened low-fat milk (*n* = 64) (18%En = 203.4 ± 53.7 g) HFCS-sweetened low-fat milk (*n* = 61) (18%En = 203.0 ± 56.9 g) fructose-sweetened low-fat milk (*n* = 65) (9%En = 171.6 ± 63.6 g) glucose-sweetened low-fat milk (*n* = 77) (9%En = 160.7 ± 51.2 g)	Hypercaloric *	Liquid	Supp/DA
Cox/Rezvani et al. ^†^ (2009) [33,34] USA	Parallel Blinded	31 overweight/obese participants (16 m/15 w) Age 40–72 years BMI 25–35 kg/m^2^	10	Intervention: fructose-sweetened beverage (*n* = 16) (175 g/day) (25%En) Control: glucose-sweetened beverage (*n* = 15) (175 g/day) (25%En)	Hypercaloric *	Liquid	Met/Supp
Jin et al. (2014) [35] USA	Parallel Double-blind Randomized	24 overweight Hispanic-American adolescents with hepatic fat > 8%, Age 11–18 years BMI ≥85th percentile	4	Intervention: fructose-sweetened beverage (*n* = 11) (99 g/day) Control: glucose-sweetened beverage (*n* = 13) (99 g/day)	Eucaloric	Liquid	Supp
Johnson et al. (2015) [40] Finland	Parallel Randomized	51 morbidly obese women with polycystic ovarian syndrome Age 18–40 years BMI ≥ 40 or 35–40 kg/m^2^	8	Intervention: moderate-fructose, low-calorie diet (LCD) (*n* = 24) (85 g fructose/day) Control: low-fructose LCD (*n* = 27) (17 g fructose/day)	Hypocaloric	Liquid	Supp/DA
Johnston et al. (2013) [37] UK	Parallel Double-blind Randomized	32 healthy overweight males Mean age 33–35 years BMI 25–32 kg/m^2^	2	Intervention: high-fructose diet (*n* = 15) (25%En = 217 g/day) Control: high-glucose diet (*n* = 17) (25%En = 215 g/day)	Eucaloric and Hypercaloric * (2 weeks each)	Liquid	Supp/Met
Lowndes et al. (2014) [43] USA	Parallel Blinded Randomized	355 overweight or obese participants (165 m/190 w) Age 20-–60 years BMI 23–35 kg/m^2^	10	Intervention: 8%En sucrose (*n* = 58) 18%En sucrose (*n* = 64)), 30%En sucrose (*n* = 53), 8%En HFCS (*n* = 69), 18%En HFCS (*n* = 60), 30%En HFCS (*n* = 51)	Hypercaloric Sugars administered in milk medium.	Liquid	Supp
Madero et al. (2011) [41] Mexico	Parallel Randomized	131 obese participants (102 w/29 m) Age 38.8 ± 8.8 years BMI 32.4 ± 4.5 kg/m^2^	6	Intervention: Low-fructose diet (<20 g/day) (*n* = 66) Control: moderate natural fructose diet (50–70 g/day) (*n* = 65)	Hypocaloric	Solid	DA
Markey et al. (2013) [45] UK	Crossover Double-blind Randomized	50 normal or overweight participants (16 m/34 w) Age 20–49 years BMI 18.5–30 kg/m^2^	8	Intervention: diet with regular sugar products (75.1 g non-milk extrinsic sugars/d) (*n* = 28) diet with sugar-reduced (reformulated) products (28.9 g non-milk extrinsic sugars/day) (*n* = 22)	Eucaloric	Mixed	Supp/DA
Raatz et al. (2015) [42] USA	Crossover Randomized	55 participants (39 w/ 16 m): group 1 with normal glucose tolerance (NGT) (*n* = 28), group 2 with impaired glucose tolerance (IGT) (*n* = 27) Mean age of NGT 39 years, Mean age of IGT 52 years BMI of NGT 26 kg/m^2^, BMI of IGT 31.5 kg/m^2^	2	Intervention: 50 g daily intake of HFCS (HFCS55) 50 g of honey 50 g of sucrose	Eucaloric	Liquid	Supp
Silbernagel et al. (2014) [36] Germany	Parallel Single-blinded Randomized	20 healthy participants (12 m/8 w) Mean age 30 years BMI of 26 ± 0.5 kg/m^2^	4	Intervention: 150 g fructose intake (*n* = 10) Control: 150 g glucose intake (*n* = 10)	Hypercaloric *	Liquid	Supp
Sorensen et al. (2005) [46] Denmark	Parallel	41 overweight participants (6 m/35 w) Age 33–37 years BMI 27–28 kg/m^2^	10	Intervention: 125–175 g/d sucrose intake (*n* = 21) artificial sweetener intake (*n* = 20)	Hypercaloric	Mixed	Supp
Yaghoobi et al. (2008) [44] Iran	Parallel Randomized	55 overweight or obese participants (24 m/31 w) Age 20-–60 years BMI > 25 kg/m^2^	≈4	Intervention: sucrose intake (70 g) (*n* = 17) honey intake (70 g) (*n* = 38)	Eucaloric	Liquid	Supp

^†^ Both studies report from one original study by Stanhope et al. [32] and each study (Cox et al., Rezvani et al.) reports on different inflammatory markers measured in the original study. ^‡^ Feeding control. Met: Metabolic feeding control was the provision of all meals, snacks, and study supplements (test sugars and foods) consumed during the study under controlled conditions. Sup: Supplement feeding control was the provision of study supplements. DA: Dietary advice is the provision of counselling on the appropriate test and control diets. § Sugar form. Dietary sugar was provided in 1 of 3 forms. Liquid: all or most of the dietary sugar was provided as beverages or crystalline sugars to be added to beverages. Solid: dietary sugar was provided as solid foods. Mixed: all or most of the dietary sugar was provided as a mix of beverages, solid foods (not fruit), and crystalline sugars. * Denotes hypercaloric studies in which fructose vs glucose interventions were administered isocalorically.

**Table 2:** Dietary intervention studies investigating the effect of fructose/HFCS, sucrose, or glucose on biomarkers of subclinical inflammation. Extracted data on baseline concentrations, results, and funding sources.

First Author, Year, Country	Outcome Baseline Concentrations ^1^	Results								Funding Source ^††^
		Percent changes in inflammatory marker after completion of intervention							Statistical Tests Comment	
		hsCRP/CRP	IL-6	TNF-α	MCP-1	sICAM-1	sE-selectin	Adipo-nectin		
Aeberli et al. (2011) [39] Switzerland	*hsCRP* (ng/mL) 205.6 ± 430.7 *Adiponectin* (μg/mL) 6.44 ± 7.69	High fructose: +109.19% * Moderate fructose:+82.2% High sucrose: +105.2% * High glucose: +89.74% *	N/A	N/A	N/A	N/A	N/A	High fructose: +18.6% * Moderate fructose: +14.8% High sucrose: +17.8% High glucose:+20.6% *	No treatment effect for hsCRP and adiponectin reported. hsCRP increased significantly after all of the interventions -highest increase observed in high fructose group	NR
Angelopoulos et al. (2016) [38] USA	*CRP* (mg/L) Fructose group: 1.74 ± 1.74 HFCS group: 1.92 ± 2.10 Sucrose group: 1.74 ± 1.78 Glucose group: 1.21 ± 1.43	Fructose: +24.1% * HFCS: −3.1% Sucrose: −1.7% Glucose: +23.9% *	N/A	N/A	N/A	N/A	N/A	N/A	No significant between-group changes in CRP for fructose, HFCS, sucrose and glucose as compared to each other. *p*-Values not reported.	Industry
Cox/Rezvani et al. (2009) [33,34] USA	*MCP-1* (pg/mL) 144.7 ± 18.8 *sE-selectin* (ng/dL) 45.0 ± 5.5 *sICAM-1* (ng/mL) 221.9 ± 6.3 *CRP* (mg/L)3.7 ± 0.8 *IL-6* (pg/mL) 3.5 ± 0.7 *Adiponectin* (ug/mL): 7.7 ± 1.1 *TNF-α*: NR	Fructose: −16.2% * Glucose: −22.8% *	Fructose: −11.4% * Glucose: +18.2% *	Fructose: −12.8% * Glucose: +0.3% *	Fructose: +37.7% * Glucose: −8.6% *	Fructose: +2.9% * Glucose: −1% *	Fructose: +14.4% * Glucose: −1.6% *	Fructose: −14.8% * Glucose: −9.1% *	Significant between-group change in MCP-1 (*p* = 0.03). Significant within-group change in sE-selectin (*p* = 0.048). But no significant between-group difference (*p* = 0.17). No significant between-group change in sICAM-1 (*p* = 0.22) CRP (*p* = 0.33) IL-6 (*p* = 0.31) adiponectin (*p* = 0.10) TNF-α (*p* = 0.42)	Agency
Jin et al. (2014) [35] USA	*hsCRP* (mg/L) 6.78 ± 3.16	Fructose: +4.13% * Glucose: −23.4% *	N/A	N/A	N/A	N/A	N/A	N/A	Significant between-group change in hsCRP (*p* = 0.019).	Agency
Johnson et al. (2015) [40] Finland	*CRP* (mg/L) Low-fructose: 6.8 ± 7.4 Moderate-fructose: 10.9 ± 10.2	Low-fructose: −8.8% Moderate-fructose: −29.3%	N/A	N/A	N/A	N/A	N/A	N/A	No significant between-group change in CRP (*p* = 0.278) Confounder (low-calorie diet → weight loss)	Agency
Johnston et al. (2013) [37] UK	*CRP* (mg/L) 1.01 ± 1.08 *IL-6* (pg/mL) 3.56 ± 4.84 *TNF-α* (pg/mL)1.92 ± 0.5	Isocaloric period: Fructose:−21.8% * Glucose: −11.4% * Hyper-caloric period: Fructose:−8.9% * Glucose:+40% *	Isocaloric period: Fructose:−4.2% * Glucose: −5.8% * Hyper-caloric period: Fructose:+23.8% * Glucose:−39.6% *	Isocaloric period: Fructose:−0.5% Glucose: −2.5% Hyper-caloric period: Fructose: −4.7% Glucose: −0.5%	N/A	N/A	N/A	N/A	No significant between-group change in CRP (*p* = 0.37), IL-6 (*p* = 0.23) or TNF-α (*p* = 0.36) in isocaloric or hypercaloric periods	Agency Industry—related conflict of interest
Lowndes et al. (2014) [38]USA	*CRP* (mg/L) HFCS 8%En intake: 1.9 ± 1.9 HFCS 18%En intake: 1.6 ± 1.6 HFCS 30%En intake: 2.1 ± 2.1 Sucrose 8%En intake: 1.5 ± 1.6 Sucrose 18%En intake: 2.0 ± 1.8 Sucrose 30%En intake: 1.5 ± 1.8	HFCS: 8%En: +26.3% 18%En: +25% 30%En: 0% Sucrose: 8%En: +40% 18%En: +5% 30%En: +6.7%	N/A	N/A	N/A	N/A	N/A	N/A	No significant between-group change (HFCS vs. sucrose) (*p* = 0.679)No significant between-group changes in CRP between various intake amounts (8% vs. 18% vs. 30%) (*p* = 0.597) Percent increases in the 30%En groups were low/lowest.	Industry
Madero et al. (2011) [41] Mexico	*sICAM* (ng/dL) Low-fructose: 4.44 ± 0.11 Moderate-fructose: 4.37 ± 0.11	N/A	N/A	N/A	N/A	Low-fructose: −6.3% Moderate-fructose: −9.6%	N/A	N/A	No significant between-group change in sICAM-1 (*P* = 0.19) Significant within-group decrease for sICAM-1 in low-fructose (*p* = 0.01) and moderate-fructose (*p* < 0.0001). Confounder (low-calorie diet → weight loss)	Agency
Markey et al. (2013) [45] UK	*CRP* (mg/L) Regular sugar intake: 0.93 ± 0.94 Reduced sugar intake: 1.05 ± 1.35	Regular sugar: +6.5% Re-formulated sugar: +15.2%	N/A	N/A	N/A	N/A	N/A	N/A	No treatment effect for sucrose (*p* = 0.593)	Agency
Raatz et al. (2015) [42] USA	*hsCRP* (mg/L) Glucose tolerant (NGT): 2.2 ± 0.5 Glucose impaired (IGT): 4.6± 0.8 *IL-6* (pg/mL) Glucose tolerant (NGT): 1.6 ± 0.2 Glucose impaired (IGT): 2.6 ± 0.5	HFCS: NGT: −5% IGT: +29.6% Sucrose: NGT: −20% IGT: +15.8% Honey: NGT: +8.7% IGT: +41.2%	HFCS: NGT: +7.7% NGT: +6.7% Sucrose: IGT: −22.2% NGT: +3.5% Honey: NGT: +23.1% IGT: +19.4%	N/A	N/A	N/A	N/A	N/A	No treatment effect for hsCRP or IL-6.	Agency
Silbernagel et al. (2014) [36] Germany	*CRP* (mg/dL) 0.13 ± 0.06 *MCP-1* (pg/mL) 275 ± 34 *E-selectin* (ng/mL) 31.8 ±5.1	Fructose: −7.7% * Glucose: +57% *	N/A	N/A	Fructose: −16.7% * Glucose: −9.1% *	N/A	Fructose: −7.8% * Glucose: +3.5% *	N/A	No significant between-group change in CRP (*P* = 0.284), MCP-1 (*p* = 0.803) or E-selectin (*p* = 0.311)	Agency
Sorensen et al. (2005) [46]Denmark	*CRP* (mg/L) 1.8 (0.9–3.0)	Sucrose: +6% Artificial sweetener: −26%	N/A	N/A	N/A	N/A	N/A	N/A	No significant between-group change (*p* = 0.1) Percent changes reported after excluding 4 subjects with CRP > 10 mg/L	Industry
Yaghoobi et al. (2008) [44] Iran	*hsCRP* (mg/dL) Healthy subjects (normal hsCRP levels): 4.8 ± 3.2 Subjects with elevated hsCRP 9.9 ± 3.6	Sucrose: −1% Honey: −3.3%	N/A	N/A	N/A	N/A	N/A	N/A	No significant between-group effect observed (*p* > 0.5).	Agency

* represents studies in which fructose or sucrose was isocalorically compared to glucose. ^1^ Data refer to mean ± SD unless otherwise indicated; N/A: not investigated NR: not reported. ^2^ Both studies report from one original study by Stanhope et al. [32] and each study (Cox et al., Rezvani et al.) reports on different inflammatory markers measured in the original study. ^††^ Funding sources. Agency: funding from government, university, or not-for-profit health agency sources. Industry: funding from companies that utilize dietary sugar for profit. NR: not reported. Johnston et al. reports conflict of interest of the author, IA Macdonald, who is on the Scientific Advisory Boards for Mars, Inc. and Coca Cola.

**Table 3:** The effects of dietary sugar on hs(CRP).

hs(CRP) (mg/L) Intervention vs. Control	No. of Studies	No. of Participants	MD	95% CI	*p*-Value	*I*^2^ (%) (95% CI) ^1^	Quality of Meta-Evidence (NutriGrade) ^2^
Fructose vs glucose	6	403	−0.03	−0.52, 0.46	(*p* = 0.90)	44 (0, 75)	Low
HFCS vs sucrose	3	677	0.21	−0.11, 0.53	(*p* = 0.19)	0 (0, 75)	Low

HFCS, high-fructose corn syrup. Pooled estimates of effect sizes (95% confidence intervals) expressed as mean differences (MD). MD = mean of intervention group—mean of control group. MD = 0: no difference between groups. MD > 0: greater value of respective outcome measured in intervention (first) groups. MD < 0: greater value of respective outcome measured in control (second) groups. ^1^ *I*^2^ value represents degree of heterogeneity within comparison groups. *I*^2^-value of greater than 50% was considered to indicate substantial heterogeneity. ^2^ Nutrigrade is an applied scoring system to judge the quality of meta-evidence [22].
